# Protocol of a monocentric, double-blind, randomized, superiority, controlled trial evaluating the effect of in-prison OROS-methylphenidate *vs.* placebo treatment in detained people with attention-deficit hyperactivity disorder (BATIR)

**DOI:** 10.1186/s13063-023-07827-7

**Published:** 2024-01-04

**Authors:** Stéphanie Baggio, Joël Billieux, Anja Dirkzwager, Katia Iglesias, Karine Moschetti, Nader Perroud, Marie Schneider, Nathalie Vernaz, Hans Wolff, Patrick Heller

**Affiliations:** 1 Institute of Primary Health Care (BIHAM), University of Bern, Bern, Switzerland; 2 Laboratory of Population Health (#PopHealthLab), University of Fribourg, Fribourg, Switzerland; 3 Division of Prison Health, Geneva University Hospitals, Geneva, Switzerland; 4 Institute of Psychology, University of Lausanne, Lausanne, Switzerland; 5 Center for Excessive Gambling, Addiction Medicine, Lausanne University Hospitals (CHUV), Lausanne, Switzerland; 6 Netherlands Institute for the Study of Crime and Law Enforcement, Amsterdam, The Netherlands; 7 School of Health Sciences, HES-SO University of Applied Sciences and Arts of Western Switzerland, Fribourg, Switzerland; 8 Health Technology Assessment Unit, University Hospital of Lausanne, Lausanne, Switzerland; 9 Center for Primary Care and Public Health (Unisanté), University of Lausanne, Lausanne, Switzerland; 10 Department of Psychiatry, Faculty of Medicine, University of Geneva, Geneva, Switzerland; 11 Division of Psychiatric Specialties, Department of Psychiatry, University Hospitals of Geneva, Geneva, Switzerland; 12 Department of Psychiatry, Dalhousie University, Halifax, Nova Scotia Canada; 13 Institute of Pharmaceutical Sciences of Western Switzerland, University of Geneva, University of Lausanne, Geneva, Switzerland; 14 Medication adherence and Interprofessionality lab, School of Pharmaceutical Sciences, University of Geneva, Geneva, Switzerland; 15 Division of Clinical Pharmacology and Toxicology, Geneva University Hospitals and Faculty of Medicine, Geneva, Switzerland

**Keywords:** Access to health care, Attention-deficit/hyperactivity disorder, Prison, Recidivism

## Abstract

**Background:**

Attention-deficit hyperactivity disorder (ADHD) is characterized by difficulty paying attention, poor impulse control, and hyperactive behavior. It is associated with several adverse health and social outcomes and leads to an increased risk of criminality and recidivism. Worldwide, ADHD is thus highly prevalent in prisons. However, ADHD treatment has been neglected in such environments. Stimulant medications such as osmotic-release oral system methylphenidate (OROS-MPH) are first-line treatments in the general population, but they are under-prescribed in prisons due to concerns about abuse, even though such claims are not empirically supported. This project aims to compare the efficacy of a 3-month in-prison OROS-MPH *vs.* placebo treatment on the severity of core ADHD symptoms and relevant in- and post-prison outcomes.

**Methods:**

This study is a phase III, double-blinded, randomized, superiority, controlled trial of OROS-MPH *vs.* placebo. After randomization, the participants will receive 3 months of treatment with OROS-MPH or placebo (1:1 ratio) while incarcerated. Upon release, all participants will be offered the treatment (OROS-MPH) for 1 year but will remain blinded to their initial study group. The study will be conducted at the Division of Prison Health, Geneva, Switzerland, among incarcerated men (*n* = 150). Measures will include (1) investigator-rated ADHD symptoms, (2) acute events collected by the medical and prison teams, (3) assessment of the risk of recidivism, (4) medication side effects, (5) medication adherence, (6) study retention, (7) health care/prison costs, and (8) 1-year recidivism. Analyses will include bivariable and multivariable modeling (e.g., regression models, mixed-effects models, survival analyses) and an economic evaluation (cost-benefit analysis).

**Discussion:**

We expect that early identification and treatment of ADHD in prison will be an important public health opportunity and a cost-effective approach that is likely to reduce the vulnerability of incarcerated individuals and promote pathways out of criminal involvement. The study will also promote standards of care for people with ADHD in prison and provide recommendations for continuity of care after release.

**Trial registration:**

ClinicalTrials.govNCT05842330. Registered on June 5, 2023.

Kofam.ch SNCTP000005388. Registered on July 17, 2023.

**Supplementary Information:**

The online version contains supplementary material available at 10.1186/s13063-023-07827-7.

## Introduction

### Background and rationale

Attention-deficit hyperactivity disorder (ADHD) is a disorder characterized by difficulty paying attention, poor impulse control, and hyperactive behavior [1]. ADHD usually begins in childhood and persists into adulthood in 40–60% of cases [2]. There is growing evidence that adult ADHD is associated with a wide range of adverse outcomes and is therefore an important public health problem [3, 4]. It affects multiple domains of life, including psychosocial functioning, school, work, and health care access and use [5–7]. It is also associated with an increased risk of having justice involvement at a younger age, including rule-breaking behavior, delinquency, crime, and recidivism [8–10]. As a result, ADHD is more prevalent in the incarcerated population than in the general population, and there is a growing recognition that ADHD in prison is a major problem [9, 11]. Several studies have been conducted to estimate the prevalence rate of ADHD in prison. A recent meta-analysis of approximately 70,000 detained persons worldwide, found an ADHD prevalence of 26.2% [11]. This is five times higher than in the general population (5%) [12, 13]. This means that ADHD should be considered as a major challenge for prison health care.

Compared with other detained persons, incarcerated individuals with ADHD experience functional impairments that are likely to interfere with prison life. For example, they are more likely to be verbally and physically aggressive and to have disciplinary infractions than detained persons without ADHD [14, 15]. They also have more unsuccessful experiences with the criminal justice system [16]. Their actions may be (mis)interpreted as intentional misbehavior rather than as features of undiagnosed ADHD [16]. In the general population, individuals usually develop coping mechanisms to deal with ADHD (e.g., preferring flexible environments with various short-term tasks and flexible deadlines). The use of such strategies is not easy in the strict prison environment, which further emphasizes the importance of access to treatment in prison [17]. A body of evidence also suggests that detained persons with untreated ADHD have high rates of recidivism [8, 18–21]. Therefore, identification and treatment of ADHD in detained persons may help prevent recidivism [22].

ADHD is a treatable condition, with pharmacotherapy being the first-line treatment [23, 24]. Psychostimulants, such as osmotic-release oral system methylphenidate (OROS-MPH) have demonstrated excellent short-term efficacy in various populations [4, 25]. Previous studies have also reported promising persistent effects after discontinuation [26, 27]. This may be due to neuroplasticity and the development of coping strategies to deal with ADHD-related impairments [27]. A combination of pharmacological and non-pharmacological treatments has been most consistently associated with long-term improvement in ADHD symptoms and global functioning [28, 29]. This is particularly true for cognitive behavioral therapy, which aims to develop behavioral strategies to compensate for the core deficits of ADHD [24, 30]. Thus, multimodal treatment is recommended for the treatment of ADHD [25].

Despite these compelling research findings, access to ADHD treatment in the adult general population remains low. ADHD is likely to be underdiagnosed and undertreated [31]. Individuals seeking diagnosis and treatment encounter barriers to care, such as waiting lists and unaware primary care professionals [31, 32]. In addition, transitions between different types of health care (e.g., from inpatient to outpatient services or from pediatric to adult health services) are a major threat to quality and continuity of care and medication therapy [33, 34]. ADHD is also likely to be underdiagnosed and undertreated in prison [35]. Stimulants such as OROS-MPH are often underprescribed in prison because of concerns about abuse [17, 36], even though such claims are not empirically supported. A primary principle of prison health care is equality of treatment [37, 38]. Prison populations should have access to and benefit from the same treatments as the general population. Restricting access to stimulant medications violates this principle. In addition, continuity of care is a major challenge for prison populations. Health care linkage programs between prison and community services are lacking, and there are several missed opportunities to treat this vulnerable population with high health needs [39–43]. A recent meta-analysis concluded that continuity of care between prison and the community should be prioritized [44]. The implementation of more programs orientated to continuity of care is needed to improve medication adherence after release. The examination and evaluation of such actions will improve knowledge on their effects.

However, to date, very few studies have investigated whether OROS-MPH is effective in prison populations [45]. To date, only three randomized control trials (RCTs) have examined the efficacy of OROS-MPH in prison populations worldwide [46–48]. Two studies concluded that it had promising positive effects, with a reduction in ADHD symptom severity [46, 47]. These preliminary findings suffer from some important methodological shortcomings that limit their generalizability, including small sample sizes (*n* = 30 and *n* = 54), high attrition rates, a focus on participants with co-occurring amphetamine use disorder, and a lack of a control group to determine post-detention treatment effects [49]. The third study examined the effects of an 8-week OROS-MPH treatment in detained young men with ADHD (aged 18–25 years) in the UK [50, 51]. ADHD symptoms did not respond to OROS-MPH. Results were also not significant for secondary outcomes (e.g., emotional dysregulation, aggression). Recidivism and adherence to treatment were not included as study outcomes in this short-term study which only takes place during imprisonment.

Two other large studies are worth considering. First, a retrospective study of Swedish population-based data found an inverse association between pharmacological treatment of ADHD (including, but not limited to, MPH) and recidivism, suggesting a protective effect [23]. The second study suggested that psychostimulants were associated with a substantial reduction in violent recidivism [52]. However, studies using individual-level data with OROS-MPH treatment and focusing on post-release outcomes are lacking. Thus, empirically based evidence on the efficacy of in-prison ADHD treatment for the rehabilitation of this population is needed [53], as well as robust evidence-based studies testing treatments that are likely to address modifiable risk factors for recidivism [44].

ADHD has a significant economic burden for the prison health care system [54, 55], but also for society, with justice-related costs being substantial [56]. Few studies have examined the costs of ADHD and ADHD treatment in prisons. To our knowledge, only one study conducted in Scotland estimated the cost of undiagnosed ADHD in the prison system [57]. It provided a conservative estimate of £590 per detained person (a total of £11.7 million per year), which included costs associated with the use of medical services and critical incidents. Although this study concluded that undiagnosed ADHD is costly to the prison system, to date, no economic evaluation has been conducted to assess the costs and the outcomes of ADHD treatment in prison. Such a study is critically needed to assess the cost-effectiveness in prison settings [58] and to provide a complete picture of its value in such settings.

### Objectives

To fill previous research gaps, we defined five sets of research objectives, related to clinical, behavioral, recidivism, economic, and side-effect outcomes.

O1. The study will compare the 3-month in-prison efficacy of OROS-MPH vs. placebo on the severity of ADHD core symptoms before release.

O2. The study will compare the 3-month in-prison efficacy of OROS-MPH vs. placebo on acute events before release.

O3. The study will compare the 3-month in-prison efficacy of OROS-MPH vs. placebo on the risk of recidivism prior to release.

O4. The study will compare the efficacy of receiving OROS-MPH vs. placebo for 3 months during incarceration on adherence to medication for the entire study period (during detention and 12 months after release).

O5. The study will compare the efficacy of receiving OROS-MPH vs. placebo for 3 months during incarceration on study retention for the entire study period (during detention and 12 months after release).

O6. The study will conduct a cost-benefit analysis of ADHD treatment (OROS-MHP *vs.* placebo) in a prison setting for two periods (during detention and 12 months after release) and according to two perspectives:

- The *health care system perspective* (third party payer): The costs and induced benefits (savings) of the ADHD treatment will be evaluated in terms of costs with respect of the health care services use (outpatient care, inpatient care, emergency interventions, and medications).
- The *prison system perspective*: Costs and induced benefits of the ADHD treatment will be evaluated in terms of costs associated with respect of the acute events related to security (e.g., sanctions) and recidivism.

O7. The study will compare the efficacy of receiving OROS-MPH vs. placebo for 3 months during incarceration on recidivism 12 months after release.

O8. The study aims to evaluate the 3-month side effects of OROS-MPH vs. placebo and its tolerability in terms of incidence of main side effects before release (Table 1).

**Table 1:** Summary of research objectives

Topic	Outcome	In prison (3 months)	Post-release (12 months)
Clinical	Severity of ADHD core symptoms	Primary objective (O1)	-
	Medication adherence	Secondary objective (O4)	
Behavioral	Acute events	Secondary objective (O2)	-
	Study retention	Secondary objective (O5)	
Recidivism	Risk of recidivism	Secondary objective (O3)	-
	One-year recidivism	-	Secondary objective (O7)
Side effects	Side effects	Secondary objective (O8)	-
Economical	Cost-benefits	Secondary objective (O6)	

### Trial design

This is a phase III double-blind, superiority, randomized, controlled trial of OROS-MPH *vs.* placebo on a clinical outcome, with a parallel design. After randomization, participants will receive 3 months of treatment with either OROS-MPH or placebo (1:1 ratio) while incarcerated. Upon release, all participants will be offered OROS-MPH treatment, without being unblinded to the treatment they received while incarcerated. They will be followed up for 12 months after release, to estimate the long-term effects of in-prison treatment (total study duration: 15 months). The participants, research team, statistician, and clinicians will be blinded to the participant group. The psychiatrist will be unblinded upon release in order to adjust the treatment according to the participant group. Participants will be unblinded at the end of the study.

## Methods: participants, interventions, and outcomes

### Study setting

Data will be collected in two prisons for which the Division of Prison Health provides health care, among men sentenced to prison in Geneva, Switzerland (Champ-Dollon and La Brenaz) (*n* = 150). The study is considered as monocentric, as the same team provides health care in both prisons. The same study team will recruit participants and collect data in both prisons. The prison population is relatively homogeneous. In these two prisons, most detained persons have short-term sentences.

### Eligibility criteria

*Inclusion criteria* are (1) age between 18 and 65 years, (2) good command of French, (3) to be released in approximately 4 months at the eligibility visit, (4) endorsement of clinical diagnostic criteria for DSM-5 ADHD, and (5) written informed consent.

*Exclusion criteria* are (1) presence of an acute uncontrolled comorbid psychiatric disorder, (2) placement in a closed center after release, (3) medical contraindication to stimulant prescription, (4) potential adverse interaction with another medication, (5) already receiving ADHD treatment, and (6) not planning to stay in Switzerland for at least 1 year.

### Informed consent

The interviewer will explain to each participant the nature of the study, its purpose, the procedures involved, the expected duration, the potential risks and benefits, and any discomfort it may cause. We will use a teach-to-goal procedure to enhance consent [59]. This process includes a comprehension test (asking questions about what participants have understood) and a retest after feedback to achieve a voluntary and truly informed consent. Each participant will be informed that study participation is voluntary, that he may withdraw at any time, and that withdrawal of consent will not affect his subsequent medical care. Participants are informed that they may ask any questions and consult with family members, friends, their treating physicians, or other experts before deciding whether to participate in the study. Participants will be given sufficient time to do so. The material for informed consent can be found in Supplementary File 1.

All participants receive a participant information sheet and a consent form that describe the study and provide sufficient information to make an informed decision about study participation. The formal consent of a participant, using the approved consent form, will be obtained before the participant undergoes any investigational procedure.

The participant should read and understand the informed consent form and voluntarily agree to sign and date the informed consent form. The informed consent form is signed and dated by the participant and the principal investigator (PI) or designees.

### Additional consent provisions for collection and use of participant data and biological specimens

An additional consent form will be proposed to study participants for reuse of their data for further research.

### Interventions

#### Intervention description

Participants will be randomized to either OROS-MPH or placebo group, stratified by prison. Participants will receive OROS-MPH (available in Switzerland as a first-line treatment for ADHD) or placebo once daily in the morning. The dosage of OROS-MPH will be determined according to the Swiss regulating authority, Swissmedic (from 18 to 72 mg/day). In both groups, the psychiatrist will start with the lowest dose (18 mg) and adjust it weekly or as needed, depending on tolerability, clinical response, and observations made by the professionals or the patient’s entourage regarding attention, impulsivity, hyperactivity, and behavioral problems. The treatment will be monitored weekly for the first month, and then monthly, except for side effects which will be monitored daily in prison and every 2 weeks after release. The psychiatrist will be unblinded when the participants are released. At this point in the study, all participants will be offered to have the treatment (OROS-MPH) without being unblinded to their initial group (OROS-MPH or placebo). Therefore, participants in the placebo group will be able to start OROS-MPH.

#### Explanation of the choice of comparator

There is no established standard of care for ADHD treatment in correctional settings. This constitutes a challenge in determining the appropriate comparator for our trial. Data on the efficacy of OROS-MPH in prison are scarce, especially including acute events in prison and post-release outcomes. To answer our research questions, the active treatment will be compared to a placebo condition, which can be considered as a ‘delayed intervention’ control. Indeed, all participants will eventually receive the active treatment, albeit at a different time (i.e., upon release for participants in the placebo group). All participants will receive a psychotherapeutic intervention (in prison and after release).

#### Criteria for discontinuation or modification of assigned interventions

For participants, the main risks are side effects of OROS-MPH, including appetite reduction, weight loss, insomnia, nervousness, mood instability, aggressiveness, agitation, anxiety, depression, irritability, abnormal behavior, headache, dizziness, dyskinesia, psychomotor hyperactivity, drowsiness, arrhythmia, tachycardia, palpitations, hypertension, cough, pharyngeal and laryngeal pain, abdominal pain, diarrhea, nausea, stomach aches and vomiting, dry mouth, alopecia, pruritus, skin rash, urticaria, arthralgia, and blood pressure change. The most common side effects are headache, appetite reduction, insomnia, anxiety, tachycardia, dry mouth, and nausea. Blood pressure and heart rate will be monitored at each dose adjustment or every 6 months. The treatment will be discontinued if participants develop symptoms (chest pain, syncope, or other symptoms suggestive of heart disease). At the psychological level, OROS-MPH may increase addictive and suicidal behavior. Changes will be monitored at each visit and we will adjust the dose accordingly. If participants experience other side effects, they will be referred for a medical evaluation and we will adjust or discontinue the treatment.

#### Strategies to improve adherence to interventions

Adherence to treatment is a critical issue for ADHD treatment and for prison populations. It is therefore defined as a study outcome (see also the “Plans to promote participant retention and complete follow-up” section).

#### Relevant concomitant care allowed or prohibited during the trial

In addition to the pharmacotherapy, both groups will receive an individualized in-prison psycho-educational program, the Young-Bramham program, provided by a trained psychologist [60, 61]. This program is a 13-session cognitive-behavioral therapy designed to provide patients a psychoeducation about ADHD and to develop specific skills for coping with difficulties related to attention, hyperactive, and impulsive symptoms. Participants will have weekly visits with the psychologist during detention. After release, they will be offered cognitive-behavioral therapy and will have visits every 2 weeks.

OROS-MPH will not be given to patients treated with monoamine oxidase (MAO) inhibitors (currently or within the last 2 weeks). There is a risk of a sudden increase in blood pressure during surgery. OROS-MPH will not be taken on the day of surgery if surgery is planned. Serotonin syndrome has already been reported after concomitant use of MPH and serotonergic medications. If a serotonin syndrome is suspected, MPH will be discontinued immediately. The safety of combining MPH on with clonidine or other centrally acting α2 agonists has not yet been systematically studied. The treatment will be discontinued if participants are receiving clonidine.

### Provisions for post-trial care

At the end of the trial, the participants will be referred to a psychiatrist for further care.

### Outcomes

#### Conners Adult ADHD Rating Scale (CAARS) [62] (O1)

We will use the short version of the observer-rated version of this scale to monitor ADHD symptoms over time (CAARS-O:S). The CAARS has shown good psychometric properties (internal consistency, convergent validity, discrimination between clinical and non-clinical groups) [63]. The CAARS has been used as the primary outcome in all prison-based RCTs [46, 47, 50]. We will use the CAARS score before intervention and at the 3-month follow-up, with the 3-month follow-up measure as the primary outcome, comparing the means between the two groups. A 1-week window period will be used (assessment between 12 and 14 weeks).

#### Acute events (O2)

Actions such as refusal to see doctors, nurses, or lawyers, hunger strikes, self-harm events or fights requiring a visit to the medical unit [64, 65], and disciplinary sanctions, including misuse of the prescribed treatment [14] will be recorded. We will use data routinely collected by the medical team and from official prison records. We will use the final number of acute events at the 3-month follow-up. We will compare the medians between the two groups.

#### Structured Assessment of Protective Factors for Violence Risk (SAPROF) (O3)

This dynamic risk assessment tool will be used to assess recidivism risk. It is a prevention-oriented assessment that identifies protective factors that may moderate risk factors and reduce the likelihood of recidivism [66]. It helps provide a balanced and comprehensive assessment of recidivism risk. The SAPROF has a good predictive value, interrater reliability, and sensitivity to treatment outcomes [67]. We will use the SAPROF score at the 3-month follow-up. To provide a reliable assessment of recidivism risk, the SAPROF must be combined with the Historical Clinical and Risk Management 20 (HCR-20), which will also be assessed in the study [68]. This structured tool assesses the risk of violence and consists of 20 items. We will compare the means between the two groups.

#### Medication adherence (O4)

The nurse will provide daily information on medication adherence during imprisonment (direct observed therapy). After release, an electronic pill bottle (Medication Event Monitoring System (MEMS), Aardex Group) will provide a sequence of binary data each day indicating whether the patient took the OROS-MPH as prescribed or not. Each month after the release, at least three trained community pharmacies, located in different areas of Geneva, will provide the OROS-MPH in MEMS bottles to participants. MEMS data will be used to derive initiation of self-management, medication implementation (quality of daily intake), and persistence on OROS-MPH after release. We will describe daily medication adherence longitudinally (yes/no) during 12-month post-release. In addition, we will use three questions to validate the use of the electronic pill bottle (how quickly participants took the medication after opening the pill bottle, whether they prepared doses of medication in advance without using the pill bottle, and whether there was a period when they did not use the pill bottle but took the medication, e.g., incarceration, hospitalization). We will compare the proportions between the two groups.

#### Retention in study (O5)

We will record dropouts from the study [47], registered using the last visit/contact with the medical unit. Reasons for dropout (if given) will be recorded. We will use the retention in the study (yes/no) at 12-month post-release. We will compare the proportions between the two groups.

#### Economic evaluation (O6)

For the *economic evaluation* (O6), we will evaluate medical and non-medical costs. Medical costs will be estimated from the perspective of the health care system and will include those of medical services used by patients (outpatient, emergency, and inpatient resources). During detention, they will be obtained from the analytical/accounting systems of the prison and the Geneva University Hospitals which is responsible for health care in prison. We will use medical costs at 3 months. Non-medical costs are prison system-related costs and will include those related to disciplinary sanctions (use of prison resources and prison staff), and recidivism-related costs (average cost of a day in prison). Information on costs will be collected prospectively during the 3 months in prison, except for recidivism, which will be collected at the end of the study. We will use prison-related costs at 3 months and 12 months after release. We will compare the medians between the two groups.

#### Recidivism (O7)

We will extract data from the official Swiss criminal records 1 year after release. The data will be requested from the Swiss Federal Office of Statistics (Criminal Conviction Statistics). Recidivism (yes/no) will be assessed 1 year after prison release. We will compare the proportions between the two groups.

#### Side effect outcomes (O8)

The main side effects will be assessed and recorded at each visit (Swismedic, see section “Criteria for discontinuation or modification of assigned interventions”). We will also record unexpected side effects. We will use side effects (yes/no and separate side effects) at the 3-month follow-up. We will compare the proportions between the two groups.

We will collect baseline variables of interest:

*Sociodemographic and prison characteristics* will include gender, age, level of education, region of origin, marital status, health insurance prior to incarceration, and reason for incarceration. We will also record whether participants are receiving in/out-patient therapeutic measures (Art. 59–63 Swiss Criminal Code).

*Medical history*, including somatic illnesses, medications, and psychiatric disorders will be assessed. We will use DSM-5 diagnoses, with the Mini-International Neuropsychiatric Interview (MINI) [69] and a scale for borderline personality disorder [1]. We will also assess impulsivity using the short impulsive behavior scale (UPPS-P) [70]. Previous ADHD treatment and use of psychotropic medication will be recorded.

### Participant timeline

#### Eligibility visit

The research and clinical teams will identify eligible detained persons, based on a list provided by the Cantonal Office of Detention). An eligibility visit will be scheduled (total duration of the visit: 1 h). During this visit, the interviewer (a trained psychologist) will assess in/exclusion criteria. A physician or psychiatrist will be involved to assess medical contraindications to stimulant prescription and potential adverse interactions. The participant will be invited to sign the informed consent and the additional consent for reuse of data collected in the study. Eligible participants diagnosed with ADHD will be referred to the psychiatrist for evaluation and appropriate mental health care.

#### Baseline assessment

Participants will meet with the interviewer for the first assessment. Participants will provide information about their medical history (including an assessment of psychiatric disorders) and sociodemographic background. The interviewer will also assess ADHD symptoms (total duration: about 1 h).

#### First visit to the psychiatrist

Participants will meet with the psychiatrist to begin the treatment (30 min).

#### In-prison treatment

Visits with the psychiatrist are scheduled to monitor the treatment (weekly for the first month, then monthly and more often as needed). Side effects and medication adherence will be recorded daily by the nurses. Weekly visits with a psychologist are scheduled to provide the 13 sessions of psychoeducation (duration: 1 h).

#### Three-month assessment

Three months after the start of the treatment, just before being released, the participants will meet with the interviewer to assess recidivism risk. The interviewer will assess ADHD symptoms.

#### Post-prison treatment

The psychiatrist will monitor the treatment monthly or more often as needed. Visits with the psychologist are scheduled every 2 weeks to provide cognitive-behavioral therapy (Table 2).

**Table 2:**
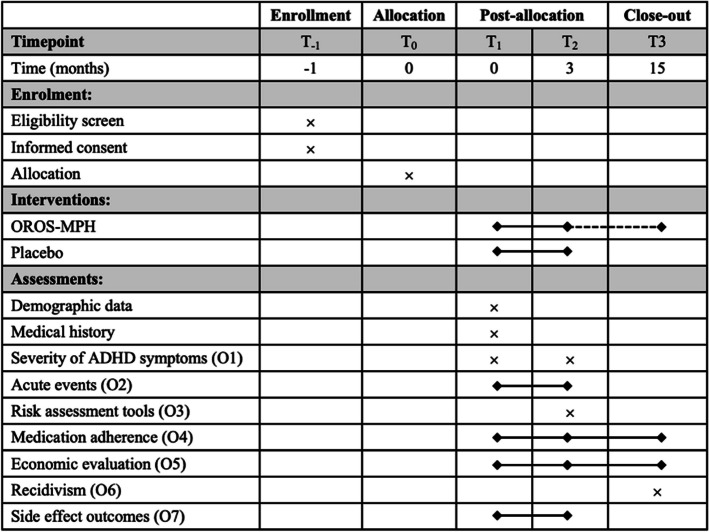
Assessment schedule

### Sample size

We calculated the sample size using G*Power 3.1, with means difference between two independent groups (*t*-test), assuming equal variances between groups. Participants will be stratified by prison, but on average, we expect no difference between participants in the two prisons. With *α* = .05, *ß* = .80, allocation 1:1, and standardized mean difference = 0.5 (average standardized effect size of 12-week treatment with OROS-MPH on change in severity of core ADHD symptoms, 25), we need *n* = 126. To account for potential attrition (e.g., participants transferred to another prison), we increased the sample to *n* = 150 (*n* = 75 in each group), which represents an attrition rate of 16%.

### Recruitment

The research and clinical teams will identify eligible detained persons based on the list provided by the cantonal prison authority. Participant recruitment may be challenging. We addressed this potential threat by using a long recruitment period (one year and a half) in two prisons. Response rates in prison studies are usually high. In a recent study conducted in Champ-Dollon [35], the response rate was 85%. However, if after 3 months we find that it is not possible to include approx. three participants per week, we will consider relaxing in/exclusion criteria to reach our primary objective (being released in approx. 4 months, stay in Switzerland for at least 1 year).

### Assignment of interventions: allocation

The pharmacy of the Geneva University Hospitals (HUG), which will prepare the medications, will randomize the sequence of treatments using a generated list (Excel macro with Visual B) and will keep the list concealed. The allocation ratio between the two arms will be 1:1. A unique randomization number will be assigned to eligible participants after the eligibility visit. The study team, who will enroll participants, will receive a sequentially numbered list. Each eligible participant will be assigned a unique randomization number corresponding to the lowest number available on the list.

### Assignment of interventions: blinding

Study participants, care providers, outcome assessors, investigators, and the study team will be blinded to group allocation. The Clinical Trial Unit of the HUG pharmacy will oversee the blinding. MPH will be supplied in colored, over-encapsulated tablets and filled with mannitol to prevent tablet movement within the capsule. The placebo will be presented in similar tablets (colored and filled with mannitol). Once prepared, they will be blistered in 7 pieces and placed in a box. When delivered to the prison, medications will be stored in the prison pharmacy. The HUG pharmacy will provide the required number of blinded kits with the randomization number. Three boxes per participant will be delivered immediately after inclusion and will be stored at the prison pharmacy. Participants will only see the encapsulated tablets and will not be able to deduce group allocation.

General unblinding will be scheduled after the final statistical analysis plan meeting, once the analyses have been validated by the investigators and sponsor. Unblinding on principal investigator (PI) request should only occur in the case of an adverse event for which it is necessary to know the study treatment in order to determine an appropriate course of therapy for the participant, by calling the HUG pharmacy. The HUG pharmacy will perform the unblinding and provide the randomization code according to the pharmacy’s current standard operating procedures. The unblinding performed by the HUG pharmacy at the request of the PI will be reported immediately by the PI to the study sponsor.

### Data collection and management

#### Plans for assessment and collection of outcomes

A description of the study instruments and their reliability and validity is given in the “Outcomes” section. Designated study team members (e.g., interviewer) will enter data into the REDCap© electronic clinical database for each enrolled participant. They will be trained. Data will be manually validated by the investigators and the central data monitor.

#### Plans to promote participant retention and complete follow-up

Retention in the study, especially after release, may be a challenge. Participants will be analyzed in their original study group (intention-to-treat analyses), except for those who explicitly withdraw their consent. If the study team has not heard from the participant, we will make every effort to contact him, determine the reason for the discontinuation of treatment, and encourage the participant to come for an end-of-study visit. If all attempts fail, the participant will be declared as “lost to follow-up”. Measures planned after prison release are not affected by attrition. For medication adherence, we will use the worst-case scenario (dropouts will be considered as non-adherence to medication). The study retention outcome will have no missing values. One-year recidivism will be obtained from the Swiss Federal Office of Statistics and is therefore expected to have no missing values. We will use the following strategies to improve study retention: [1] train the interviewer to locate and contact participants who drop out [71] and [2] use compensation to encourage in-prison participation [72]. We also increased the sample size by 16% to have sufficient power to draw conclusions regarding our primary outcome.

#### Data management

A certified GCP-compliant electronic clinical data management system (REDCap©) will be used. REDCap© allows specific role distinction, personal identification of authorized users, automatic reporting, monitoring, and data safety. Actions are reported in a detailed audit trail system. Data hosted in the system are coded, so that data entries cannot be identified. While data are on REDCap©, they will be hosted at the University of Bern. Central data monitoring will be performed by the study team and supervised by Prof. Katia Iglesias (expert in clinical trials). Data management will be performed by the interviewer/coordinator.

#### Confidentiality

A limited number of persons will have access to the participants’ personal details (investigators, interviewer, psychologist, and psychiatrist). To ensure confidentiality, this electronic list will be kept in a separate password-protected file. The signed informed consents will be locked in a cabinet in the PI’s office. Direct access to source documents will be allowed for monitoring, auditing, and inspection purposes.

Data will be stored on the University’s secure servers. We will use unique and persistent identifiers. Data will be publicly available (but only upon request) and only human subject data that is properly anonymized and prepared according to applicable legal and ethical guidelines will be entered in the online database. At the end of the study, data (dataset and documentation) will be archived and stored online in a repository (SWISSUbase). All information will be available in this repository. A confirmation from the sponsor will be used to access data, as the data are sensitive. A non-disclosure agreement will provide adequate protection for confidential data. Data will be available as soon as the results are published. Recidivism data will not be available (sensitive data category 3 according to the Swiss Federal Office of Statistics).

#### Plans for collection, laboratory evaluation, and storage of biological specimens for genetic or molecular analysis in this trial/future use

Not applicable.

### Statistical methods

#### Statistical methods for primary and secondary outcomes

##### O1

We will use a linear regression model (or negative binomial, depending on the outcome’s distribution) to test whether ADHD treatment (OROS-MPH *vs.* placebo) predicts the severity of core ADHD symptoms.

##### O2

We will use a negative binomial regression model to test whether ADHD treatment (OROS-MPH *vs.* placebo) predicts the number of acute events. Sensitivity analyses will include a logistic regression model with the presence or absence of acute events and a logistic regression model focusing on treatment misuse.

##### O3

We will use a linear regression model to test whether ADHD treatment (OROS-MPH *vs.* placebo) predicts the recidivism risk.

##### O4

The repeated, correlated adherence measurements will be described longitudinally using generalized estimating equations models. We will use Cox regression to test whether ADHD treatment (OROS-MPH *vs.* placebo) predicts adherence to medication: (1) whether people have started their OROS-MPH on the own after prison release (called “initiation”); (2) whether medication implementation trajectories (daily medication intake) differ between groups at 3, 6, and 12 months post-incarceration; (3) if persistence (time between initiation and discontinuation) to OROS-MPH differs between groups (event: stop medication on his own without a shared decision with physician or study dropout). We will also test whether ADHD treatment (OROS-MPH *vs.* placebo) predicts the decision to take OROS-MPH after release. A systematic computerized term search in the adherence report will be performed to identify the most frequent variables affecting adherence and a regression model will be used to discover their association with adherence.

##### O5

We will use Cox regression to test whether ADHD treatment (OROS-MPH *vs.* placebo) predicts study retention (event: study dropout).

##### O6

For the economic evaluation, cost-benefit analyses will be performed and aggregated outcomes will be calculated. We will use regression models to estimate differences in costs across OROS-MPH and placebo groups adjusting for covariates. Because data will probably be skewed, we will use gamma generalized linear models with a log link. We will perform analyses separately for prison and health care costs. The net benefit (differences) and/or benefit-to-cost ratio generated by the intervention will be reported using means and 95% confidence intervals. No discount rate will be applied given that the time horizon will not be larger than 15 months. Uncertainty will be assessed using univariate and probabilistic sensitivity analyses.

##### O7

We will use Cox regression to test whether ADHD treatment (OROS-MPH *vs.* placebo) predicts recidivism (event: first recidivism). We will consider alternative outcome measures in sensitivity analyses (e.g., severity of recidivism, time to event).

##### O8

We will calculate descriptive statistics for each type of side effect, the presence of any side effect, and the sum of side effects. We will compare the two groups (OROS-MPH vs. placebo) using bivariate analyses (logistic and negative binomial regressions).

We will use a two-sided *α* = .05 for all statistical analyses. We will use a Bonferroni-Holms adjustment in case of multiple testing. Statistical software will include Stata 18 and R version 4 (or new versions of these statistical software). All analyses will be conducted as intention-to-treat. Participants will be analyzed in their original study group irrespective of the treatment they received, except for those who explicitly withdraw their consent. For all objectives, we will perform sensitivity analyses controlling for baseline covariates and including the stratification factor [73], following European Medicines Agency (EMA) guidelines [74]. Alternatively, we will also perform a series of sensitivity analyses using an inverse probability weighting strategy to adjust for baseline covariates [75, 76].

### Interim analyses

The data collection will end when all data have been collected, with no planned interim analyses.

### Methods for additional analyses (e.g., subgroup analyses)

Not applicable.

### Methods in analysis to handle protocol non-adherence and any statistical methods to handle missing data

Deviations from the original statistical plan will be reported and justified in the final report. They will be reported as post-hoc analyses.

We will examine the distributional properties of the variables, correlations, and patterns of missing data for self-reported data. In the case of missing values or dropouts, we will use inverse probability attrition weighting to correct our estimates for attrition. This method has recently been successfully used in RCTs [77, 78].

### Plans to give access to the full protocol, participant-level data, and statistical code

The full protocol, anonymized dataset, and statistical code will be available in a Findable, Accessible, Interoperable, and Re-usable (FAIR) repository (SWISSUbase).

### Oversight and monitoring

#### Composition of the coordinating center and trial steering committee

The sponsor is Prof. Stéphanie Baggio. The investigators are Prof. Stéphanie Baggio and Dr. Patrick Heller. The statisticians are Prof. Stéphanie Baggio and Prof. Katia Iglesias. Prof. Katia Iglesias is also the Central Data Monitor. The monitoring institution is the Clinical Trial Unit (CTU) of the University of Bern, Switzerland. The steering committee is composed of members of the Division of Prison Health, Geneva University Hospitals, Switzerland: Madalena Almeida (secretary), Prof. Stéphanie Baggio (researcher, sponsor, co-PI), Dr. Komal Chacowry Pala (physician), Diane Golay (psychologist, head of psychologists), Dr. Laurent Gétaz (physician, head of Champ-Dollon prison), Dr. Leonel Gonçalves (researcher, study coordinator), Dr. Patrick Heller (psychiatrist, PI and head of psychiatrists), Prof. Katia Iglesias (trialist), Miriam Kasztura (nurse), Nicolas Peigné (nurse), and Prof. Hans Wolff (physician, head of the Division). Meetings are held regularly every 2 months. Prof. Marie Schneider is responsible for the measure of participants’ medication adherence in selected community pharmacies in Geneva during the 12-month post-incarceration monitoring. The Clinical Trials Unit of the Geneva University Hospitals Pharmacy, Switzerland, is in charge of the preparation of medications during incarceration and randomization. After release, the medication will be provided by pharmacists and invoiced to health insurance.

#### Composition of the data monitoring committee, its role, and reporting structure

This is a monocentric, investigator-initiated trial. The local ethics committee did not require a data monitoring committee, as this is a low-risk intervention. Interim analyses are not required for patient protection.

#### Adverse event reporting and harms

Adverse events (AEs) and serious adverse events (SAEs) will be collected during 3 months in prison. AEs include the main side effects from Swissmedic information. They will be assessed at each visit, using open-ended questions. Participants will also be able to report AEs to the prison clinical team. The following main side effects will be non-systematically assessed and recorded at each visit: Appetite reduction, weight loss, insomnia, nervousness, mood instability, aggressiveness, agitation, anxiety, depression, irritability, abnormal behavior, headache, dizziness, dyskinesia, psychomotor hyperactivity, drowsiness, arrhythmia, tachycardia, palpitations, hypertension, cough, pharyngeal and laryngeal pain, abdominal pain, diarrhea, nausea, stomach aches and vomiting, dry mouth, alopecia, pruritus, skin rash, urticaria, arthralgia, and blood pressure change. Adverse events will be reported without a specific threshold. Time of onset, duration, resolution, action taken, assessment of severity, and relationship to study treatment will be recorded. Other unexpected adverse events will be recorded using the open-ended format of the questions. AEs will be discussed with the clinical team (psychologists and psychiatrists). All SAEs will be reported to the Sponsor-Investigator of the study within 24 h. SAEs resulting in death will be reported to the Ethics Committee within 7 days and a pharmacovigilance report will be written and submitted to the pharmacovigilance authorities.

#### Frequency and plans for auditing trial conduct

Monitoring visits to the study site will be organized by the Clinical Trial Unit of the University of Bern. This will ensure that the trial is conducted in accordance with Good Clinical Practices recommendations, protocol instructions, and amendments (if applicable) and that severe adverse events are adequately reported. We plan three site monitoring visits: (1) at the beginning of the study (after inclusion of 2–3 participants); (2) in the middle of the study, and (3) at the end of the study. The process will be independent from the investigators and the sponsor. The sponsor will be responsible of the site initiation visit. The study documents and source data/documents will be available to the auditors/inspectors, the ethic committee, and the cantonal authorities, and questions will be answered during inspections. All parties involved must maintain the strict confidentiality of the participants’ data.

#### Plans for communicating important protocol amendments to relevant parties

Sponsor and PIs are allowed to amend the protocol or to make suggestions for protocol amendment. Substantial protocol amendments (e.g., changes in eligibility criteria, outcomes, analyses) will be communicated to competent authorities. Substantial amendments will be implemented only after the approval of the competent authorities. In emergency situations, deviations from the protocol to protect the rights, safety, and well-being of human participants may be implemented without prior approval of the sponsor and competent authorities. Such deviations must be documented and reported to the sponsor and competent authorities as soon as possible. All non-substantial amendments will be communicated to the competent authorities as soon as possible.

#### Dissemination plans

Results will be presented at international scientific conferences and articles will be published in leading peer-reviewed journals. Authors of the core study include the investigators, project partners, and the research team: Stéphanie Baggio, Joël Billieux, Leonel da Cunha Gonçalves, Anja Dirkzwager, Katia Iglesias, Patrick Heller, Karine Moschetti, Nader Perroud, Elena Poznyak, Melina Romao dos Santos, Marie Schneider, Nathalie Vernaz, and Hans Wolff. Additional publications are expected to result from the study. We will establish a consortium to include members of the Division of prison health involved in the study. We will follow authorship guidelines, including (1) substantial contribution to the conception, design, acquisition, analysis, or interpretation of the study; (2) drafting or critically revising the manuscript; (3) final approval of the manuscript; and (4) being accountable for all aspect of the work. We also plan to disseminate results at the prison and health care levels, to promote access to ADHD treatment in prison.

## Discussion

This study will contribute to advance knowledge on ADHD treatment at multiple levels. These findings are expected to have a high impact for both research and clinical practice.

At the *individual level*, the study will show how to improve the health of detained persons, including access to timely and appropriate ADHD treatment and continuity of care. Treatment of ADHD is essential for promoting desistance [79]. Indeed, ADHD is critical in the criminal career, both for initial arrest and recidivism.

First, this study will contribute to advance research in the field of prison mental health, with valuable insights into the benefits of treating ADHD during detention. It includes [1] evidence on a reduction in the severity of core ADHD symptoms in this vulnerable population often having comorbidities and lacking access to health care and [2] a reduction in problematic behaviors that may lead to sanctions. Second, the study will produce important findings on the effects of in-prison treatment *after release*. The post-prison part of the study will examine important aspects of ADHD treatment: Continuity of care and recidivism. These questions are critical for detained persons with ADHD. Indeed, patients with ADHD are likely to have difficulties with engagement and anticipation.

At the *clinical level*, the study will help to understand if prison, a controlled setting where detained persons can receive health care and support from health care professionals, is a good setting to start ADHD treatment. Our study will inform on the long-term effects of OROS-MPH, even if treatment is discontinued. The main goal for ADHD treatment should be not only to temporarily reduce symptoms, but also to contribute to a more favorable life trajectory after incarceration [80]. Our study has the potential to improve the identification and treatment of ADHD in prison, which will be seen as an important public health opportunity, able to reduce the vulnerability of detained persons and promote desistance. We will also be able to provide empirically based recommendations for the treatment and management of ADHD in prison.

At the *prison level*, our study will improve safety for those who work and live in prison. By increasing adjustment to prison life and reducing rule-breaking behaviors and confrontations with the prison staff or other detained persons, it will improve safety and prison climate. It will also help to increase the awareness of ADHD and mental health care in prison and its benefits.

At the *society/public health level*, by evaluating the costs and benefits of ADHD treatment, our study will help to reduce health care use and consequently associated costs, with a better health care for ADHD. Our study will also inform how continuity of care can be achieved, thus providing guidance on how to improve the transition between prison and community health services. It will show whether the prison setting is an opportunity to diagnose and treat the unmet health needs of ADHD and to prioritize care for it. Another public health benefit will be to provide estimates of recidivism for detained persons with ADHD in prison, as data on this topic are severely lacking. Potential benefits also include improvements in medical practice and the future development of appropriate public health planning.

## Trial status

This manuscript refers to version 5 of the protocol (October 13, 2023). Participant recruitment will start in November 2023 and is planned to be completed by May 2025. The study is expected to be completed by September 2026. The trial is registered on ClinicalTrials.gov (ID NCT05842330, registered on June 5, 2023) and Kofam.ch (ID SNCTP000005388, registered on July 17, 2023). We used the Standard Protocol Items: Recommendations for Interventional Trials (SPIRIT) checklist (Supplementary File 2) and the World Health Organization Trial Registration Data Set (Supplementary File 3).

### Supplementary Information

**Additional file 1.** Informed consent.

**Additional file 2.** SPIRIT 2013 Checklist: Recommended items to address in a clinical trial protocol and related documents.

**Additional file 3.** World Health Organization Trial Registration Data Set.

## Data Availability

The Case Report Form (French version) is available upon request to the corresponding author.

## Abbreviations

ADHD: Attention-deficit hyperactivity disorder
HUG: Geneva University Hospitals
OROS-MPH: Osmotic-release oral system methylphenidate
PI: Principal Investigator
