# Supplementary material for: Protocol of a monocentric, double-blind, randomized, superiority, controlled trial evaluating the effect of in-prison OROS-methylphenidate vs. placebo treatment in detained people with attention-deficit hyperactivity disorder (BATIR)
Source: Trials. 2024 Jan 4;25:23. doi: 10.1186/s13063-023-07827-7 (PMC10765778; doi:10.1186/s13063-023-07827-7)
Supplement: Supplementary file 1 — Additional file 1. Informed consent. [file 13063_2023_7827_MOESM1_ESM.pdf]

Hôpitaux
Universitaires
Genève

Demande de participation à un projet de recherche médical :

Essai clinique contrôlé randomisé pour le traitement du trouble du déficit d’attention avec ou sans hyperactivité en prison

Monsieur,

Nous vous proposons de participer à notre étude.

Votre participation est entièrement libre. Toutes les données collectées dans le cadre de ce projet sont soumises à des règles strictes en matière de protection des données.

Le projet de recherche est mené par Prof. Stéphanie Baggio et Dr. Patrick Heller. Nous vous en communiquerons les résultats si vous le souhaitez.

Lors d’un entretien, nous vous présenterons les éléments essentiels et répondrons à vos questions. Pour vous proposer d’ores et déjà un aperçu du projet, voici les points clés à retenir. Vous trouverez à la suite des informations complémentaires plus détaillées.

Pourquoi menons-nous ce projet de recherche ?

- En présence d’un trouble du déficit d’attention avec ou sans hyperactivité (TDAH), on procède à un traitement médicamenteux à base de méthylphénidate (Concerta®) en complément d’un suivi psychothérapeutique, dans le but d’améliorer l’attention et de diminuer l’agitation.
- Notre projet de recherche vise à étudier si l’efficacité du Concerta® associé à un suivi psychothérapeutique est supérieure à un suivi psychothérapeutique seul.

Que dois-je faire si j’accepte de participer ? – Que se passe-t-il pour moi en cas de participation ?

- **Forme de la participation** : Si vous acceptez de participer à notre étude, vous recevrez un traitement médicamenteux à base de méthylphénidate (Concerta®) pour soigner le TDAH ou un placebo et vous aurez un suivi psychothérapeutique.
- **Déroulement pour les participants** : Les participants sont répartis au hasard dans 2 groupes.
    - Groupe 1 : Vous recevez la substance testée (Concerta®) en prison. Vous décidez si vous voulez continuer le traitement après libération. Vous recevez un suivi psychothérapeutique en prison et après libération.
    - Groupe 2 : Vous recevez le placebo en prison. Vous décidez si vous voulez recevoir la substance testée (Concerta®) au moment de la libération. Vous recevez un suivi psychothérapeutique en prison et après libération.
- **Durée** : 15 mois (3 mois pendant l’incarcération et 12 mois après la libération).

- **Nombre de consultations, temps nécessaire et contraintes associées** :
  - Entretiens avec un chercheur : 3 entretiens (avant l'inclusion dans l'étude, au début de l'étude et après 3 mois) (durée 1 heure chacun).
  - Consultations avec une psychologue : toutes les semaines pendant l'incarcération et toutes les deux semaines après libération (durée 1 heure chacune).
  - Consultations avec un psychiatre : au début de l'étude pour commencer le traitement, puis une fois par semaine le premier mois pour adapter le traitement, ensuite une fois par mois pour le suivi du traitement et au moment de la libération pour évaluer la poursuite du traitement (durée 30 minutes chacune).
  - Le médicament vous sera donné tous les jours par l'équipe infirmière en prison et remis toutes les deux semaines dans une pharmacie de Genève (dans une des 4 pharmacies partenaires de l'étude) après libération.

Quels sont les bénéfices et les risques liés à la participation au projet ?

Bénéfices pour les participants

- La participation à ce projet peut vous apporter un bénéfice direct. Cela permettra de diagnostiquer et de traiter votre TDAH. Le traitement permet d'améliorer l'attention et de diminuer l'agitation. La psychothérapie vous permettra de mieux gérer votre TDAH au quotidien.
- Par votre participation, vous contribuez à aider les personnes atteintes de ce trouble et à améliorer les connaissances et l'accès au traitement pour les personnes incarcérées.
- Vous recevrez CHF 100.- de dédommagement pour votre participation : CHF 50.- à la première visite (T_1) avec le chercheur (avoir pour l'épicerie de la prison) et CHF 50.- à la seconde visite avec le chercheur (T_2) (bon Migros).

Risques et contraintes

- Le Concerta® peut avoir des effets secondaires :
  - Très fréquents : nervosité, insomnie, maux de tête, réduction de l'appétit.
  - Fréquents : vertiges, dyskinésie (mouvements involontaires), hyperactivité psychomotrice, somnolence, troubles gastro-intestinaux (douleurs abdominales, diarrhée, nausées, maux d'estomac et vomissements) survenant en début de traitement et qui peuvent être atténués par la prise de nourriture en même temps que le médicament, sécheresse de la bouche, réactions cutanées (alopécie, prurit, éruption cutanée, urticaire).
  - Occasionnels : perte de poids, instabilité de l'humeur, agressivité, agitation, anxiété, dépression, irritabilité, comportement anormal, modifications de la fréquence cardiaque (arythmie, tachycardie, palpitations), modification de la tension artérielle, hypertension, toux, douleurs pharyngées et laryngées et douleurs articulaires.

En apposant votre signature à la fin du document, vous certifiez en avoir compris tout le contenu et consentir librement à prendre part au projet.

Information détaillée

1. Objectif du projet et sélection des participants

Dans cette feuille d'information, notre projet de recherche est désigné par le terme *étude*. Si vous acceptez d'y prendre part, vous êtes *un participant à l'étude*.

Cette étude doit nous permettre d'examiner et de mesurer l'efficacité du médicament Concerta® dans le traitement du trouble du déficit d'attention avec ou sans hyperactivité (TDAH) chez les personnes en détention. Ce médicament contient le principe actif appelé méthylphénidate et présente les propriétés suivantes : il aide à augmenter l'attention et à diminuer l'agitation des personnes pour lesquelles un médecin a posé le diagnostic de TDAH. Il s'agit d'un médicament à libération prolongée, c'est-à-dire qu'il se diffuse lentement et longtemps dans l'organisme.

Nous vous sollicitons car la participation est ouverte à tous les adultes en détention avec un diagnostic de TDAH.

2. Informations générales sur le projet

Les effets bénéfiques du Concerta® chez les enfants et les adultes sont bien connus. En revanche, les études manquent en prison. Le TDAH est rarement diagnostiqué en prison, et quand il l'est, il est peu traité à cause de craintes que le traitement soit détourné (revendu ou mal utilisé). De plus, nous ne savons pas si le traitement permet de réduire les problèmes en prison (bagarres, sanctions, etc.) et après libération (récidive).
Nous souhaitons donc évaluer les bénéfices du Concerta® pour les adultes incarcérés.

Si vous participez au projet, vous serez affecté au hasard dans un des deux groupes :
- Groupe 1 : Vous recevez la substance testée (Concerta®) en prison. Vous décidez si vous voulez continuer le traitement après libération. Vous recevez un suivi psychothérapeutique en prison et après libération.
- Groupe 2 : Vous recevez le placebo en prison. Vous décidez si vous voulez recevoir la substance testée (Concerta®) au moment de la libération. Vous recevez un suivi psychothérapeutique en prison et après libération.

Vous ne saurez pas si vous avez reçu le Concerta® ou le placebo en prison avant la fin de l'étude, c'est-à-dire 12 mois après libération.

Le Concerta® est un médicament autorisé en Suisse pour le traitement du TDAH. Il s'agit d'un comprimé à avaler une fois par jour. Nous utilisons les dosages recommandés en Suisse. Le traitement commence à la dose minimale de 18 mg/jour, qui sera adaptée par le psychiatre chaque semaine ou selon les besoins. En général, la dose est augmentée de 18 mg/jour chaque semaine.

Il s'agit d'une étude qui se déroule uniquement à Genève, aux prisons de Champ-Dollon et La Brenaz. 150 participants y prendront part. Votre participation à l'étude durera 15 mois (3 mois en prison, 12 mois après libération).

Actuellement, le TDAH n'est pas diagnostiqué et très rarement traité dans les prisons de Genève. Quand il est traité, les patients reçoivent du Concerta®.

Cette étude est réalisée dans le respect des prescriptions de la législation suisse. Nous suivons en outre l'ensemble des directives reconnues au niveau international. La commission d'éthique compétente et Swissmedic ont examiné et autorisé l'étude.

Vous trouverez également un descriptif de l'étude sur le site Internet de l'Office fédéral de la santé publique : www.kofam.ch (SNCTP000005388).

3. Déroulement pour les participants

L'étude prévoit :

- Trois entretiens avec un chercheur (durée environ 1 heure chacun) :
  - Avant l'inclusion dans l'étude, pour vérifier si vous pouvez y participer et vous proposer de signer le consentement (T_0). A la fin de cet entretien, vous rencontrerez également un médecin pour s'assurer que vous n'avez pas de problème médical qui vous empêche de recevoir le médicament. Le médecin vérifiera avec vous les maladies que vous pouvez avoir et les médicaments que vous prenez. Il effectuera un électrocardiogramme. Si nécessaire, des examens complémentaires (bilan en cas de maladie cardiaque, dosage TSH en cas de maladie thyroïdienne) seront effectués ;
  - Au début de l'étude, pour vous poser des questions sur vous (informations socio-démographiques), votre parcours médical et évaluer la sévérité de votre TDAH (T_1) ;
  - Après 3 mois et avant votre libération, pour évaluer de nouveau la sévérité de votre TDAH et faire une évaluation du risque de récidive (T_2).

- Des consultations avec une psychologue (durée 1 heure chacune) :
  - Toutes les semaines pendant l'incarcération, pour un suivi psychothérapeutique vous permettant d'apprendre à mieux gérer les symptômes du TDAH et pour le suivi du traitement (oublis, problèmes, effets secondaires, etc.) ;
  - Toutes les deux semaines après libération, pour un suivi psychothérapeutique et le suivi du traitement.

- Des consultations avec un psychiatre (durée environ 30 minutes) :
  - Au début de l'étude, pour commencer le traitement ;
  - Toutes les semaines le premier mois pour adapter le traitement, ensuite tous les mois pour le suivi du traitement ;
  - A la libération, pour évaluer et poursuivre le traitement ;
  - Tous les mois après libération pour le suivi du traitement.

- Le médicament vous sera remis :
  - Tous les jours par l'équipe infirmière en prison ;
  - Toutes les deux semaines après libération, dans une pharmacie de Genève (dans une des 4 partenaires de l'étude). Le médicament vous sera remis dans un pilulier électronique, qui enregistrera les dates et heures d'ouverture.

L'étude prévoit également de collecter les informations sur les coûts médicaux et la récidive. Pour cette partie, nous n'avons pas besoin de vous poser de questions ou de vous recontacter. L'administration fédérale fournira des informations sur la récidive de tous les participants de l'étude une fois celle-ci terminée. L'information sera fournie de manière anonyme. Cela signifie que la récidive (ou son absence) ne pourra en aucun cas vous être attribuée personnellement.

Schéma de votre participation dans l'étude

	En prison			Après libération
	Avant l'étude (T_0)	Début de l'étude (T_1)	A 3 mois (T_2)	
**Entretiens** avec le chercheur	Evaluation critères d'inclusion (1h)<br>- Diagnostic du TDAH<br>- Questionnaire d'inclusion dans l'étude<br>- Consentement	Questionnaire début d'étude (1h)<br>- Sévérité du TDAH<br>- Informations socio-démographiques<br>- Parcours médical	Questionnaire fin d'étude (1h)<br>- Sévérité du TDAH<br>- Evaluation risque de récidive	
**Consultation** avec le médecin	Evaluation critères d'inclusion (20 min)<br>- Mesures médicales			
**Consultations** avec le psychiatre		Initiation du traitement (30 min)<br>Puis selon besoin ou tous les mois	Evaluation et poursuite du traitement (30 min)	Selon besoin ou tous les mois
**Consultations** avec la psychologue		Suivi psychothérapeutique et suivi du traitement<br>Puis chaque semaine		Toutes les 2 semaines
**Médicament** Infirmier/pilulier		Donné tous les jours par l'équipe infirmière		Délivré toutes les 2 semaines par la pharmacie dans un pilulier

Il se peut que nous devions arrêter votre participation à l'étude avant le terme prévu. Cette situation peut se produire si vous supportez mal le traitement. Dans ce cas, nous vous proposerons pour votre propre sécurité de vous examiner une dernière fois. Après libération, nous vous demanderons de nous rapporter tous les médicaments qui vous ont été remis. La poursuite de votre prise en charge médicale est assurée en tout temps. Si vous arrêtez le traitement, vous continuerez à bénéficier du suivi psychothérapeutique jusqu'à la fin de l'étude.

4. Bénéfices pour les participants

Si vous participez à l'étude, cela permettra de diagnostiquer et traiter votre TDAH. Cela pourra vous aider à améliorer votre attention, diminuer votre agitation et mieux gérer votre TDAH au quotidien. Mais il se peut aussi que vous ne tiriez aucun bénéfice de votre participation. Les résultats de l'étude pourraient se révéler importants par la suite pour les personnes détenues ayant un TDAH.

5. Caractère facultatif de la participation et contraintes

Votre participation est entièrement libre. Si vous choisissez de ne pas participer ou si vous choisissez de participer et revenez sur votre décision pendant l'étude, vous n'aurez pas à vous justifier. Cela ne changera rien à votre prise en charge médicale habituelle.

Si vous choisissez de participer à l'étude, vous serez tenu de :

- Suivre les instructions et de remplir les exigences prévues par le protocole de l'étude ;
- Informer le médecin-investigateur de l'évolution du TDAH et de lui signaler tout nouveau symptôme, tout nouveau trouble et tout changement dans votre état ;
- Informer le médecin-investigateur de tout traitement ou thérapie prescrit par d'autres médecins, ainsi que de tous les médicaments que vous prenez.

6. Risques et contraintes pour les participants

Le Concerta® peut présenter des effets secondaires :

- Très fréquents : nervosité, insomnie, maux de tête, réduction de l'appétit ;

- Fréquents : vertiges, dyskinésie (mouvements involontaires), hyperactivité psychomotrice, somnolence, troubles gastro-intestinaux (douleurs abdominales, diarrhée, nausées, maux d'estomac et vomissements) survenant en début de traitement et qui peuvent être atténués par la prise de nourriture en même temps que le médicament, sécheresse de la bouche, réactions cutanées (alopécie, prurit, éruption cutanée, urticaire) ;
- Occasionnels : perte de poids, instabilité de l'humeur, agressivité, agitation, anxiété, dépression, irritabilité, comportement anormal, modifications de la fréquence cardiaque (arythmie, tachycardie, palpitations), modification de la tension artérielle, hypertension, toux, douleurs pharyngées et laryngées et douleurs articulaires.

7. Alternatives

La participation à l'étude présente des bénéfices et des risques. Le TDAH peut être traité avec d'autres médicaments qui ne sont pas à base de méthylphénidate (par exemple, le Strattera®, à base d'atomoxétine). Néanmoins, ces traitements sont moins efficaces. Le médecin-investigateur vous conseillera à ce sujet lors de l'entretien.

8. Résultats

L'étude permet d'obtenir différents résultats :

1. Des résultats individuels qui vous concernent directement ;
2. Des résultats définitifs de l'étude.

1. Le médecin-investigateur vous avisera pendant l'étude de tout élément nouveau importante vous concernant. Vous serez informé oralement et par écrit ; vous pourrez par la suite à nouveau décider si vous souhaitez poursuivre votre participation à l'étude.
2. Le médecin-investigateur peut vous faire parvenir, à l'issue de l'étude, une synthèse des résultats globaux.

9. Confidentialité des données et des échantillons

9.1. Traitement et codage des données

Dans le cadre de cette étude, des données relatives à votre personne et à votre santé sont recueillies et traitées. Ces informations sont codées au moment du relevé. Le codage signifie que toutes les données permettant de vous identifier (nom, date de naissance, etc.) sont remplacées par un code. Il n'est pas possible de relier les données à votre personne sans le code, qui reste en permanence au sein de l'hôpital.
Seul un nombre limité de personnes peut consulter vos données sous une forme non codée, et ce, exclusivement afin de pouvoir accomplir des tâches nécessaires au déroulement de l'étude. Ces personnes sont tenues au secret professionnel. En tant que participant, vous avez le droit de consulter vos données.

9.2. Protection des données et des échantillons

Toutes les directives relatives à la protection des données sont rigoureusement respectées. Il est possible que vos données doivent être transmises sous forme codée, par exemple pour une publication, et qu'elles puissent être mises à la disposition d'autres chercheur.e.s.

9.3. Protection des données en cas de réutilisation

Vos données et échantillons pourraient ultérieurement se révéler importants pour répondre à d'autres questionnements et/ou être envoyés à une autre banque de données/biobanque située en Suisse ou à l'étranger pour être aussi exploités dans d'autres projets de recherche (réutilisation). Cette banque de données/biobanque doit toutefois obéir aux mêmes normes et exigences que la banque de données/biobanque de la présente étude. Pour cette réutilisation, nous vous prions de signer une déclaration de consentement séparée à la fin de cette feuille d'information. Ce deuxième consentement est indépendant de la participation à l'étude.

9.4. Droit de consultation dans le cadre d'inspections

L'étude peut faire l'objet d'inspections. Celles-ci peuvent être effectuées par la commission d'éthique compétente, par l'autorité suisse de contrôle et d'autorisation des produits thérapeutiques Swissmedic ou par le promoteur qui a initié l'étude. Les investigateurs doivent alors communiquer vos données pour les besoins de ces inspections. Toutes les personnes impliquées sont tenues au plus strict secret professionnel.

10. Retrait du projet

Vous pouvez à tout moment vous retirer de l'étude si vous le souhaitez. Cependant, les données médicales recueillies jusque-là pourront encore être analysées, toujours sous forme codée.
En cas de retrait, vos données et vos échantillons continuent de figurer sous forme codée dans les documents de l'étude, en premier lieu pour assurer la sécurité médicale. Vous devez donc être d'accord avec cela avant de donner votre consentement.

11. Dédommagement

Vous percevrez le dédommagement suivant pour votre participation à cette étude :

- CHF 50.- après la participation à l'entretien d'entrée dans l'étude avec le chercheur (T_1), sous la forme d'un avoir pour l'épicerie de la prison ;
- CHF 50.- après la participation à l'entrée de 3 mois avec le chercheur (T_2), sous la forme d'un bon pour la Migros.

Votre participation n'aura aucune conséquence financière pour vous ou pour votre assurance-maladie.

12. Responsabilité

Les Hôpitaux Universitaires de Genève (promoteur) qui ont initié l'étude et sont chargés de sa réalisation, sont responsables des dommages que vous pourriez subir en relation avec la substance à l'étude ou avec les activités de recherche (p. ex. examens). Les conditions et la procédure sont fixées par la loi. Les Hôpitaux Universitaires de Genève ont conclu une assurance auprès de la compagnie Bâloise Assurance (Aeschengraben 21, 4002 Bâle) pour être en mesure de réparer les dommages relevant de leur responsabilité.
Pour les dommages occasionnés par un médicament autorisé et employé selon les standards médicaux ou qui seraient survenus lors d'un traitement avec une thérapie conventionnelle, les règles de responsabilité applicables sont celles régissant les traitements en dehors d'une étude.
Si vous subissiez un dommage du fait de votre participation à l'étude, il vous faudrait vous adresser aux investigateurs ou à l'assurance mentionnée ci-avant.

13. Financement

L'étude est intégralement financée par le Fonds National Suisse. Le Fonds National Suisse est indépendant de l'industrie pharmaceutique et finance des projets de recherche visant à améliorer les connaissances scientifiques.

14. Interlocuteur(s)

Vous pouvez à tout moment poser des questions au sujet de l'étude. En cas de doutes, de craintes ou d'urgences pendant ou après l'étude, vous pouvez vous adresser à l'un des interlocuteurs suivants :

Nom de la promotrice et co-investigatrice principale : Prof. Stéphanie Baggio, Chemin du Petit Bel Air 2, 1226 Thônex, +41 22 305 52 18, stephanie.baggio@hcuge.ch
Nom du médecin-investigateur : Dr. Patrick Heller, Chemin du Petit Bel Air 2, 1226 Thônex, +41 22 305 52 18, patrick.heller@hcuge.ch

15. Glossaire (termes nécessitant une explication)

- Placebo
  Certaines personnes à qui on donne un médicament ne recouvrent pas la santé grâce à ce médicament, mais grâce au bien que leur procure l'attention de leur médecin. On se rend bien compte de ce phénomène quand on voit certains patients guérir alors qu'on leur a remis un pseudo-médicament, c'est-à-dire qui a l'apparence d'un vrai médicament, qui présente le même emballage, mais qui ne contient en réalité aucun principe actif. C'est ce type de pseudo-médicament qu'on appelle « placebo ».
  Lors d'un essai clinique, il arrive qu'on décide de traiter une partie des participants avec le vrai médicament (contenant le principe actif) et une autre partie avec un placebo (sans principe actif). On peut ainsi comparer et mieux évaluer si les améliorations observées sont réellement dues au médicament étudié, autrement dit si celui-ci est réellement efficace, ou si ces améliorations sont à mettre sur le compte de l'attention accordée au patient ou à l'évolution naturelle de la maladie.

- Promoteur
  Le promoteur est une personne ou une institution ayant son siège ou une représentation en Suisse qui prend l'initiative d'une étude, c'est-à-dire qui porte la responsabilité de son lancement, de sa gestion et de son financement dans ce pays.

Hôpitaux Universitaires Genève

Déclaration de consentement

Déclaration de consentement écrite pour la participation à une étude clinique
Veuillez lire attentivement ce formulaire. N'hésitez pas à poser des questions lorsque vous ne comprenez pas quelque chose ou que vous souhaitez avoir des précisions. Votre consentement écrit est nécessaire pour participer au projet.

**Numéro BASEC du projet de recherche (après soumission à la commission d'éthique compétente) :**	
**Titre (scientifique et usuel) :**	Essai clinique contrôlé randomisé pour le traitement du trouble du déficit d'attention avec ou sans hyperactivité en prison
**Institution responsable (promoteur et adresse complète) :**	PD Dr. Stéphanie Baggio, Service de Médecine Pénitentiaire, Hôpitaux Universitaires de Genève, chemin du Petit Bel Air 2, 1226 Thônex
**Lieu de réalisation :**	Puplinge
**Médecin-investigateur responsable sur le site :** Nom et prénom en caractères d'imprimerie :	Dr. Patrick Heller
**Participant :** Nom et prénom en caractères d'imprimerie : Date de naissance :	

- Je déclare avoir été informé, oralement et par écrit, des objectifs et du déroulement de l'étude mettant en œuvre la substance testée ainsi que des avantages et des inconvénients possibles et des risques éventuels.
- Je prends part à cette étude de façon volontaire et j'accepte le contenu de la feuille d'information qui m'a été remise. J'ai eu suffisamment de temps pour prendre ma décision.
- J'ai reçu les réponses aux questions que j'ai posées en lien avec ma participation à cette étude. Je conserve la feuille d'information et reçois une copie de ma déclaration de consentement écrite.
- J'ai été informé des alternatives thérapeutiques au projet, p. ex. de l'existence d'autres traitements et thérapies.
- En cas de traitement ultérieur en dehors du lieu de réalisation de cette étude, j'autorise mes médecins à fournir au médecin-investigateur les données post-traitement pertinentes pour l'étude.
- J'accepte que les spécialistes compétents du promoteur de cette étude, de la commission d'éthique compétente et de l'autorité suisse de contrôle et d'autorisation des produits thérapeutiques Swissmedic puissent consulter mes données non codées afin de procéder à des contrôles et des inspections, à condition toutefois que la confidentialité de ces données soit strictement assurée.
- Je serai informé des résultats ayant une incidence directe sur ma santé. Si je ne souhaite pas obtenir ces informations, je prends contact avec le médecin-investigateur.
- Je sais que mes données personnelles, mes données de santé (et mes échantillons) peuvent être transmis(es) à des fins de recherche dans le cadre de cette étude et uniquement sous une forme codée (aussi à l'étranger). Le promoteur assure une protection des données conforme aux normes et exigences suisses.

- Je peux, à tout moment et sans avoir à me justifier, révoquer mon consentement à participer à l'étude, sans que cette décision n'ait de répercussions défavorables sur la suite de ma prise en charge. Les données qui ont été recueillies jusque-là seront cependant analysés sous forme codée dans le cadre de l'étude.
- Je suis informé qu'une assurance a été souscrite par les Hôpitaux Universitaires de Genève pour couvrir les dommages imputables au projet que je pourrais subir.
- Je suis conscient que les obligations mentionnées dans la feuille d'information destinée aux participants doivent être respectées pendant toute la durée de l'étude. Le médecin-investigateur peut m'exclure à tout moment de l'étude dans l'intérêt de ma santé.

Lieu, date	Signature du participant

Attestation du médecin-investigateur : Par la présente, j'atteste avoir expliqué au participant la nature, l'importance et la portée de l'étude. Je déclare satisfaire à toutes les obligations en relation avec cette étude conformément au droit suisse en vigueur. Si je devais prendre connaissance, à quelque moment que ce soit durant la réalisation de l'étude, d'éléments susceptibles d'influer sur le consentement du participant à prendre part au projet, je m'engage à l'en informer immédiatement.

Lieu, date	Nom et prénom de la personne habilitée à signer<br><br>Signature du membre de l'équipe de recherche, au nom du principal investigateur

Hôpitaux Universitaires Genève

Déclaration de consentement écrite pour la réutilisation de données et d'échantillons biologiques sous une forme codée

**Numéro BASEC de l'étude (après soumission à la commission d'éthique compétente) :**	
**Titre (scientifique et usuel) :**	Essai clinique contrôlé randomisé pour le traitement du trouble du déficit d'attention avec ou sans hyperactivité en prison
**Participant :**<br>Nom et prénom en caractères d'imprimerie :<br>Date de naissance :	

J'accepte que mes données obtenues dans le cadre de cette étude puissent être réutilisées à des fins de recherche médicale sous forme codée. Cela signifie que les données seront conservées dans une biobanque et ultérieurement exploitées pour une durée indéfinie dans le cadre de futurs projets de recherche.

Je sais que mes données sont conservées sous forme codée et que la liste d'identification est gardée dans un lieu sûr. Les données peuvent être envoyées à des fins d'analyse à une autre banque de données/biobanque située en Suisse ou à l'étranger, à condition qu'elle obéisse à des normes et exigences au moins équivalentes aux normes et exigences suisses. Toutes les dispositions légales relatives à la protection des données sont respectées.

Je donne mon accord de façon volontaire et je peux à tout moment revenir sur ma décision. Si je reviens sur ma décision, mes données seront rendues anonymes. Je dois simplement en informer le médecin-investigateur. Je n'ai pas à justifier ma décision.

J'accepte que mes données et mes échantillons soient anonymisés et j'ai compris que je ne pourrai pas être informé des résultats ni retirer mon consentement ultérieurement.

Je renonce à tout droit d'exploitation commerciale sur mes données.

Lieu, date	Signature du participant

Attestation du médecin-investigateur : Par la présente, j'atteste avoir expliqué au participant la nature, l'importance et la portée de la réutilisation des données.

Lieu, date	Nom et prénom de la personne habilitée à signer<br><br>Signature du membre de l'équipe de recherche, au nom du principal investigateur
