# Supplementary material for: Protocol of a monocentric, double-blind, randomized, superiority, controlled trial evaluating the effect of in-prison OROS-methylphenidate vs. placebo treatment in detained people with attention-deficit hyperactivity disorder (BATIR)
Source: Trials. 2024 Jan 4;25:23. doi: 10.1186/s13063-023-07827-7 (PMC10765778; doi:10.1186/s13063-023-07827-7)
Supplement: Supplementary file 3 — Additional file 3. World Health Organization Trial Registration Data Set. [file 13063_2023_7827_MOESM3_ESM.docx]

**Supplemental Table 2: World Health Organization Trial Registration Data Set.**

| **Item** | **Information** |
| --- | --- |
| Primary registry and trial identifying number | ClinicalTrials.gov ID: NCT05842330 |
| Date of registration in primary registry | June 5, 2023 |
| Secondary identifying numbers | SNCTP000005388 (kofam.ch), 212581 (Swiss National Science Foundation) |
| Source(s) of monetary or material support | Swiss National Science Foundation |
| Primary sponsor | University of Bern |
| Secondary sponsor (s) | Not applicable |
| Contact for public queries | Prof. Stéphanie Baggio (Principal investigator, University of Bern) stephanie.baggio@unibe.ch |
| Contact for scientific queries | Prof. Stéphanie Baggio (Principal investigator, University of Bern) stephanie.baggio@unibe.ch |
| Public title | **B**enefits of **A**DHD **T**reatment **I**n **R**ehabilitation (BATIR) |
| Scientific title | Benefits of in-prison OROS-methylphenidate *vs.* placebo treatment in detained people with attention-deficit/hyperactivity disorder: A randomized controlled trial |
| Countries of recruitment | Switzerland |
| Health condition(s) or problem(s) studied | Attention Deficit Hyperactivity Disorder (ADHD) |
| Intervention(s) | Pharmaceutical intervention*:* Osmotic-release oral system methylphenidate  Control intervention: Placebo composed of mannitol capsules |
| Key inclusion and exclusion criteria | Inclusion criteria: Age 18-65, good command of French, going to be released in approx. 4 months at eligibility visit, fulfilling clinical diagnostic criteria for ADHD, providing written informed consent.  Exclusion criteria: Presence of an acute uncontrolled comorbid psychiatric disorder, going in a closed centre after release, medical contraindication to stimulant prescription, adverse interaction with another medication, already receive ADHD treatment, do not plan to stay in Switzerland for at least one year. |
| Study type | Type of study: interventional  Method of allocation: randomized in a ratio of 1:1 in two arms  Masking: quadruple (participant, investigator, statistician, study team) |
| Date of first enrolment | November 15, 2023 |
| Target sample size | 150 participants |
| Recruitment status | Not recruiting |
| Primary outcome(s) | Observer-rated version of the *Conners Adult ADHD Rating Scale* |
| Key secondary outcomes | Acute events  Risk of recidivism (S*tructured Assessment of Protective Factors for Violence Risk*)  Medication adherence  Study retention  Economic evaluation (health care/prison costs)  One-year recidivism  Medication side effects (safety outcome) |
